# Breaking the mold: atypical lactate and ketone elevations in thyroid storm

**DOI:** 10.1210/jcemcr/luag113

**Published:** 2026-04-29

**Authors:** Nudar Bhuiya, Baby Vasanthi Kodali, Sapna Shah, Ruba Riachy

**Affiliations:** Department of Internal Medicine, MetroHealth Medical Center, Case Western Reserve University, 2500 MetroHealth Drive, Cleveland, OH 44109, USA; Department of Internal Medicine, MetroHealth Medical Center, Case Western Reserve University, 2500 MetroHealth Drive, Cleveland, OH 44109, USA; Department of Internal Medicine, Division of Endocrinology and Metabolism, MetroHealth Medical Center, Case Western Reserve University, 2500 MetroHealth Drive, Cleveland, OH 44109, USA; Department of Internal Medicine, MetroHealth Medical Center, Case Western Reserve University, 2500 MetroHealth Drive, Cleveland, OH 44109, USA; Department of Internal Medicine, Division of Endocrinology and Metabolism, MetroHealth Medical Center, Case Western Reserve University, 2500 MetroHealth Drive, Cleveland, OH 44109, USA; Department of Internal Medicine, MetroHealth Medical Center, Case Western Reserve University, 2500 MetroHealth Drive, Cleveland, OH 44109, USA; Department of Internal Medicine, Division of Endocrinology and Metabolism, MetroHealth Medical Center, Case Western Reserve University, 2500 MetroHealth Drive, Cleveland, OH 44109, USA

**Keywords:** thyroid storm, lactate, ketones, heart failure, thyrotoxicosis

## Abstract

Thyroid storm is a life-threatening condition characterized by excessive thyroid hormone activity, potentially leading to multiorgan failure. This case report describes a 67-year-old male with an unusual presentation of thyroid storm: elevated lactate and ketone levels, and acute heart failure. It highlights the importance of recognizing and promptly treating atypical manifestations to prevent adverse outcomes.

## Introduction

Thyroid storm is a decompensated state of severe thyrotoxicosis characterized by excessive thyroid hormone activity, which exceeds metabolic demands and leads to multiorgan dysfunction [1]. Prompt recognition and intervention are crucial to reducing mortality risk. Common precipitants of severe thyrotoxicosis include thionamide withdrawal, infections, acute myocardial infarction, surgery, diabetic ketoacidosis, and trauma [2]. The diagnosis is primarily clinical, supported by features of sympathetic nervous system activation, elevated thyroid hormone levels, and suppressed thyroid-stimulating hormone level [2]. Scoring systems, including the Burch–Wartofsky Point Scale and Japanese Thyroid Association scale, help predict the likelihood and severity of thyroid storm in patients with biochemical hyperthyroidism. However, they do not account for atypical clinical and laboratory findings. We report a rare presentation of thyroid storm with elevated plasma lactate and ketone levels, who also developed acute heart failure, complicating management.

## Case presentation

A 67-year-old male with a history of chronic obstructive pulmonary disease, schizophrenia, past alcohol and cocaine abuse, in remission since 2022, presented with an unintentional 20-pound weight loss over ten months, palpitations, dyspnea, fatigue, poor oral intake, and intermittent diarrhea. He had no personal or family history of thyroid disease.

## Diagnostic assessment

**Figure 1:**
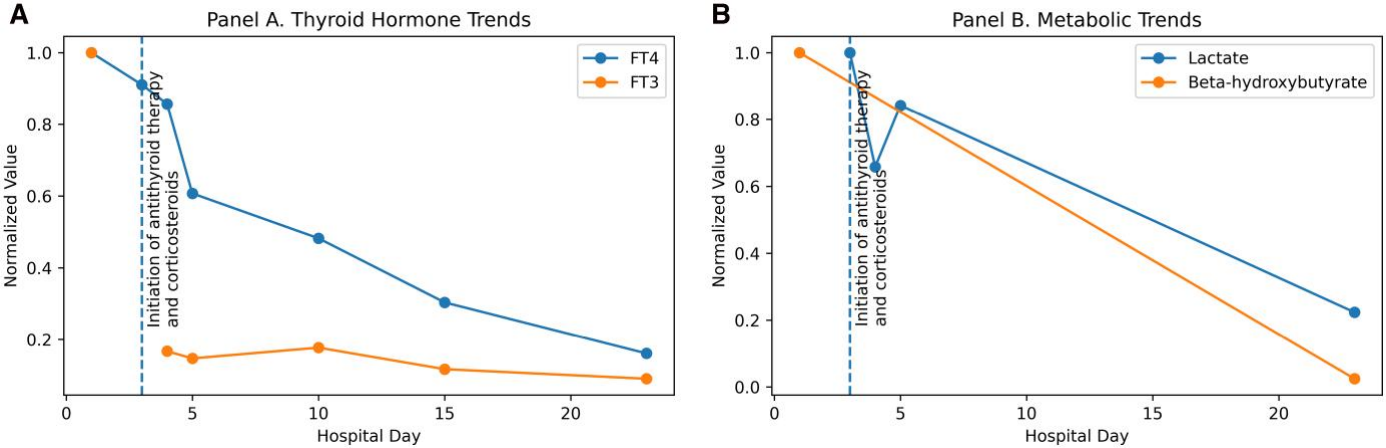
Trends in thyroid hormones, lactate, and beta-hydroxybutyrate during hospitalization following initiation of pharmacologic therapy. Normalized values of free triiodothyronine (FT3), free thyroxine (FT4), lactate, and beta-hydroxybutyrate are shown over time. Arrows indicate the initiation of hydrocortisone and methimazole therapy.

On arrival, the patient was afebrile, hypertensive (151/84 mmHg), and tachycardic (150 beats/min). He had no goiter or pertinent eye exam findings. The heart exam showed a normal rate and rhythm with no murmurs. He had bilateral rhonchi on lung exam, but no lower extremity edema. Laboratory findings revealed thyroid-stimulating hormone (TSH) <0.010 µIU/mL (<0.01 mIU/L) (reference range: 0.45-5.3 μIU/mL; 0.45-5.3 mIU/L), free thyroxine (FT4) > 5.6 ng/dL (> 72.07 pmol/L) (reference range: 0.45-1.8 ng/dL; 12-22 pmol/L), and free triiodothyronine (FT3) > 30 pg/mL (> 46.1 pmol/L) (reference range: 2.5-3.9 pg/mL; 3.5-6.5 pmol/L), Additionally, elevated lactate 2.7 mmol/L (24.33 mg/dL) (reference range: 0.5-2.2 mmol/L; 4.5-19.8 mg/dL) and beta-hydroxybutyrate 4.13 mmol/L levels(42.95 mg/dL) (reference range: <0.5 mmol/L; < 5.2 mg/dL) were noted at presentation. Both lactate and beta-hydroxybutyrate levels were checked in the setting of high anion gap, poor oral intake, and weight loss. High sensitivity troponin level was 6 ng/L (0.006 ng/mL) (reference range <15 ng/L; <0.015 ng/mL). Other labs included creatinine 0.46 mg/dL (40.66 µmol/L) (reference range: 0.7-1.3 mg/dL; 62-115 µmol/L), alkaline phosphatase 76 U/L (reference range 30-120 U/L), alanine aminotransferase (ALT) 35 U/L (reference range: 10-55 U/L), aspartate aminotransferase (AST) 42 U/L (reference range: 10-40 U/L), anion gap 25 mEq/L (reference range 8-12 mEq/L), venous blood gas pH 7.366 (reference range: 7.31-7.41). He had elevated TSH receptor antibody 2.91 IU/L (reference ≤2.00 IU/L) and thyroid-stimulating antibody 264% (reference <140% baseline) suggestive of Graves disease. Table 1 and Fig. 1 describe the changes in laboratory values during the hospital admission. Thyroid ultrasound revealed increased vascularity with high Doppler flow and bilateral thyroid nodules. The Burch-Wartofsky score was 35, indicating an impending thyroid storm.

**Table 1:** Improvement in laboratory parameters and heart rate in response to pharmacologic treatment during hospitalization

Variables	Reference range	Day 1	Day 3	Day 4	Day 5	Day 10	Day 15	Day 19	Day 20	Day 23
FT4	0.45-1.8 ng/dL (12-22 pmol/L)	>5.60 ng/dL (>72.1 pmol/L)	5.1 ng/dL (65.6 pmol/L)	4.8 ng/dL (61.8 pmol/L)	3.4 ng/dL (43.8 pmol/L)	2.7 ng/dL (34.7 pmol/L)	1.7 ng/dL (21.9 pmol/L)			0.9 ng/dL (11.6 pmol/L)
FT3	2.5-3.9 pg/mL (3.5-6.5 pmol/L)	>30.0 pg/mL (>46.1 pmol/L)		5.0 pg/mL (7.7 pmol/L)	4.4 pg/mL (6.8 pmol/L)	5.3 pg/mL (8.2 pmol/L)	3.5 pg/mL (5.4 pmol/L)			2.7 pg/mL (4.2 pmol/L)
TSH	0.45-5.3 uIU/mL (0.45-5.3 mIU/L)	<0.010 uIU/mL (<0.01 mIU/L)								
Heart Rate	60-100 bpm	150 bpm	130 bpm	83 bpm	82 bpm	101 bpm	71 bpm	50 bpm	53 bpm	53 bpm
Lactate	0.5-2.2 mmol/L (4.5-19.8 mg/dL)		7.6 mmol/L (79.04 mg/dL)	5.0 mmol/L (45.05 mg/dL)	6.4 mmol/L (57.66 mg/dL)				1.7 mmol/L (15.3 mg/dL)	
Beta- hydroxybutyrate	<0.5 mmol/L (<5.2 mg/dL)	4.13 mmol/L (42.95 mg/dL)						0.1 mmol/L (0.9 mg/dL)		
Methimazole	—		60 mg/day	60 mg/day	60 mg/day	60 mg/day	60 mg/day	60 mg/day		20 mg/day
Hydrocortisone	—		150 mg/day	150 mg/day	150 mg/day	100 mg/day	150 mg/day	150 mg/day		150 mg/day
Propranolol	—	20 mg/day	100 mg/day	120 mg/day	120 mg/day	80 mg/day	120 mg/day	120 mg/day		120 mg/day

Abbreviations: FT3, free triiodothyronine; FT4, free thyroxine; TSH, thyrotropin or thyroid-stimulating hormone; bpm, beats per minute.

## Treatment

Scheduled doses of methimazole and propranolol were initiated on presentation (refer to Table 1 for medication dose adjustments during the hospital course). Despite initial treatment, the patient experienced clinical deterioration, marked by hypotension with blood pressure of 80/65 mmHg, persistent tachycardia with heart rate of 170 beats/min, and transient non-bloody diarrhea. Blood pressure was supported by intravenous fluids. Lactate level increased to 7.6 mmol/L (79.04 mg/dL) (reference range: 0.5-2.2 mmol/L; 4.5-19.8 mg/dL), and high-sensitivity troponin rose dramatically from 106 ng/L (0.106 ng/mL) to a peak of 876 ng/L (0.876 ng/mL) (reference range <15 ng/L; <0.015 ng/mL), indicating significant myocardial stress. Echocardiography revealed a severely reduced left ventricular ejection fraction (LVEF) of 15%, with basal contraction and apical akinesia suggestive of stress cardiomyopathy.

In response to worsening symptoms, methimazole and propranolol doses were escalated, potassium iodide oral solution (SSKI) was added, and intravenous hydrocortisone was introduced to mitigate the systemic effects of excessive thyroid hormones (Table 1, Fig. 1).

## Outcome and follow-up

Given the patient's unstable social circumstances, a definitive treatment approach was pursued with a total thyroidectomy on day 24 of the same hospital admission. Following surgery, treatment including methimazole, SSKI, and hydrocortisone was discontinued. Thyroid hormones, lactate, and ketone levels normalized, and repeat echocardiography demonstrated full recovery of LVEF to 60%

## Discussion

The BWPS is a validated and widely used tool for assessing the severity of hyperthyroidism and predicting the likelihood of a thyroid storm with a score >45 being highly suggestive [3, 4]. In this case, a score of 35 due to tachycardia (25 points) and diarrhea (10 points) indicated an impending thyroid storm.

Thyroid hormones increase metabolic rate, driving energy demand and glucose utilization [5]. Hyperthyroidism stimulates hormone-sensitive lipase, causing lipolysis and elevated free fatty acid levels that undergo aerobic beta oxidation in the liver, yielding acetyl-CoA [6]. Excess acetyl-CoA is redirected toward ketogenesis when the tricarboxylic acid cycle (TCA) capacity is exceeded [6]. Simultaneously, hyperthyroidism accelerates anaerobic glycolysis in skeletal muscle, producing lactate as a byproduct. Lactate is transported to the liver for gluconeogenesis via the Cori cycle (lactic acid cycle) [5]. In severe hyperthyroidism, this accelerated Cori cycle, combined with impaired hepatic clearance, can result in lactate accumulation [5]. The accumulation of plasma lactate and ketone levels indicates significant metabolic stress, potential multi-organ dysfunction, and an increased risk of mortality [7].

The prevalence of metabolic complications such as lactic acidosis and ketoacidosis in the context of thyroid storm remains poorly defined. Available data are largely limited to isolated case reports, suggesting that while these disturbances are uncommon, they can manifest during severe thyrotoxic episodes.​

For instance, Izumi et al reported a 50-year-old woman who presented with confusion, tachycardia, and heart failure. Laboratory analysis revealed significant lactic acidosis (lactic acid concentration of 6.238 mmol/L), despite normothermia and normotension [8]. Similarly, Al-Mashdali et al described a 41-year-old woman who presented with severe abdominal pain and weight loss. Laboratory findings indicated high anion gap metabolic acidosis, positive urinary ketones, and elevated serum β-hydroxybutyrate levels with normal blood glucose and glycated hemoglobin levels. Subsequent thyroid function tests confirmed primary hyperthyroidism and non-diabetic ketoacidosis [9].

These cases highlight the under-recognition of metabolic complications in thyroid storm, possibly due to their atypical presentations and the infrequency of routine monitoring for such disturbances. Clinicians should maintain a high index of suspicion for metabolic derangements in patients with thyrotoxicosis, even when classic signs of thyroid storm are absent.

Thyrotoxic cardiomyopathy, another notable complication in this patient, is a potentially reversible condition upon achieving an euthyroid state [10]. Hyperthyroidism increases cardiac tissue sensitivity to catecholamines, causing tachycardia, increased contractility, and a high-output cardiac state that may progress to ventricular dilatation and heart failure if not treated promptly [11]. Elevated lactate and ketones exacerbate systemic acidosis, further impairing cardiac function [12].

Thyrotoxic crisis management focuses on inhibiting thyroid hormone synthesis, blocking peripheral thyroid hormone action, and addressing systemic complications [1]. Initial treatment includes thionamides, beta blockers, and glucocorticoids. Thionamides reduce thyroid hormone synthesis by inhibiting the thyroid peroxidase enzyme; propylthiouracil additionally inhibits peripheral conversion of T4 to T3 [13]. Beta blockers provide symptomatic control; high-dose propranolol additionally reduces T4 to T3 conversion, independent of beta-adrenergic receptor blockade [13]. The initial administration of propranolol in this patient may have contributed to clinical deterioration in the setting of a new heart failure diagnosis. Glucocorticoids reduce peripheral conversion of T4 to T3 and thyroxine production, especially when hyperthyroidism is secondary to Graves disease [13]. In this patient, methimazole, propranolol, and hydrocortisone led to clinical and biochemical improvement. Total thyroidectomy was necessary to ensure definitive treatment, given the patient's unstable social circumstances.

## Learning points

- Elevated plasma lactate and ketone levels, though unconventional, may signal severe metabolic derangements in thyrotoxicosis. Their recognition underscores the importance of considering nontraditional markers in complex presentations.
- Thyrotoxic cardiomyopathy, driven by heightened cardiac sensitivity to catecholamines and increased workload, exemplifies the potential for profound but reversible cardiac dysfunction with timely therapeutic intervention.
- While tools like the Burch-Wartofsky Point Scale aid in risk assessment, they may not capture the full spectrum of clinical and laboratory abnormalities. A holistic, detail-oriented approach remains critical to effective diagnosis and management.
- Recognition of atypical findings, comprehensive clinical evaluation beyond scoring systems, and aggressive treatment are critical to optimize patient outcomes.

## Contributors

All authors made substantial contributions to this work. R.R. and B.V.K. were involved in the diagnosis of the patient. R.R., B.V.K., and S.S. were involved in the management of the patient. R.R., N.B., and B.V.K. contributed to drafting the manuscript. R.R., S.S., and N.B. critically revised the manuscript for important intellectual content. All authors reviewed and approved the final version of the manuscript.

## Data Availability

Original data generated and analyzed during this study are included in this published article.
